# Extracting Robust Biomarkers From Multichannel EEG Time Series Using Nonlinear Dimensionality Reduction Applied to Ordinal Pattern Statistics and Spectral Quantities

**DOI:** 10.3389/fphys.2020.614565

**Published:** 2021-02-01

**Authors:** Inga Kottlarz, Sebastian Berg, Diana Toscano-Tejeida, Iris Steinmann, Mathias Bähr, Stefan Luther, Melanie Wilke, Ulrich Parlitz, Alexander Schlemmer

**Affiliations:** ^1^Max Planck Institute for Dynamics and Self-Organization, Göttingen, Germany; ^2^Institute for the Dynamics of Complex Systems, Georg-August-Universität Göttingen, Göttingen, Germany; ^3^Department of Cognitive Neurology, University Medical Center Göttingen, Göttingen, Germany; ^4^Department of Neurology, University Medical Center Göttingen, Göttingen, Germany; ^5^Institute of Pharmacology and Toxicology, University Medical Center Göttingen, Göttingen, Germany; ^6^German Center for Cardiovascular Research (DZHK), Partner Site Göttingen, Göttingen, Germany; ^7^German Primate Center, Leibniz Institute for Primate Research, Göttingen, Germany

**Keywords:** EEG - Electroencephalogram, t-SNE (t-distributed stochastic neighbor embedding), ordinal pattern statistics, nonlinear dimensionality reduction, biomarkers, functional connectivity, coherence, mutual information

## Abstract

In this study, ordinal pattern analysis and classical frequency-based EEG analysis methods are used to differentiate between EEGs of different age groups as well as individuals. As characteristic features, functional connectivity as well as single-channel measures in both the time and frequency domain are considered. We compare the separation power of each feature set after nonlinear dimensionality reduction using t-distributed stochastic neighbor embedding and demonstrate that ordinal pattern-based measures yield results comparable to frequency-based measures applied to preprocessed data, and outperform them if applied to raw data. Our analysis yields no significant differences in performance between single-channel features and functional connectivity features regarding the question of age group separation.

## 1. Introduction

The study of physiological networks is of great interest in biomedical sciences. Especially functional brain networks, extracted from MRI- or EEG-recordings, are a frequent subject of studies. We obtain functional networks from EEG recordings and compare these networks to features of single EEG channels in their function as biomarkers.

Neurobiological changes in healthy and pathological aging and their electrophysiological correlates (EEG) are still a hot topic in the neuroscience community, particularly since the incidence and prevalence of mild cognitive impairment and dementia increase alongside life expectancy (Ricci, 2019). Although some consensus has been reached regarding some electrophysiological correlates of aging, such as the reduction of occipital alpha power (Babiloni et al., 2006) and a shifting of the individual alpha peak towards lower frequencies in elderly subjects (Scally et al., 2018), an electrophysiological marker with which we can confidently discriminate between young and elderly individuals is yet to be established.

Thus, the study of age group differences in EEG recordings has been of interest for several decades and has been addressed by many authors over the years. While some considered differences in single-channel (SC) measures (Waschke et al., 2017), others used functional connectivity (FC) (McIntosh et al., 2013) or a combination of multiple feature groups (Al Zoubi et al., 2018). All methods successfully extracted significant differences between two age groups or significant correlations between the measure of choice and age. This is why we wanted to directly compare the discriminating power of FC and SC features in this study.

In recent years, studies aiming not only at differentiating between age groups but also between individuals based on features extracted from EEG recordings have become available (Rocca et al., 2014; Demuru and Fraschini, 2020; Suetani and Kitajo, 2020; Wilaiprasitporn et al., 2020). In all mentioned works, the features of choice were based in the frequency domain.

A problem for commonly used features extracted from EEG signals is the observation that in most cases, extensive preprocessing of the data is required and done, which has recently been shown to possibly lead to different results (Robbins et al., 2020). Thus, a method of feature extraction where the amount of preprocessing can be reduced would be desirable. Therefore, we used ordinal pattern (OP) statistics (Bandt and Pompe, 2002) for characterizing our data which has been shown to be a robust method for analysing physiological time series (Keller et al., 2007a, 2014; Parlitz et al., 2012; Amigó et al., 2015; Unakafov, 2015). For example, OP analysis has been used to separate healthy subjects from patients suffering from congestive heart failure (Parlitz et al., 2012) or to differentiate between different experimental conditions in EEG recordings (Unakafov, 2015; Quintero-Quiroz et al., 2018).

The discriminating power of OP distributions of single channels is compared to FC measures given by the mutual information (MI) based on OP distributions. These time domain features are compared to spectral features given by power spectral densities (PSDs) of single channels and coherence characterizing interrelations of pairs of channels. As a benchmark task we aim at separating individuals and age groups based on different sets of features extracted from EEG recordings. To illustrate and quantify the separation the high dimensional features (feature vectors) are mapped to a two-dimensional plane using the nonlinear dimensionality reduction algorithm t-SNE (van der Maaten and Hinton, 2008).

## 2. Materials

### 2.1. Data Set

The data set analyzed in this study was on a set of recordings from 45 participants, divided into two different age groups, who participated in an image recognition task. We will refer to this data set as the Image Recognition data set.

Twenty-two young (12 female, mean age: 24.8 years ± 3.9 SD) and 23 elderly (11 female, mean age: 62.4 years ± 7.2 SD) healthy subjects were included. All subjects had normal or corrected-to-normal vision, normal contrast sensitivity and no color vision weakness/blindness. None of the participants had a history of neurological or psychiatric diseases. Normal visual acuity, contrast sensitivity and color vision were corroborated with the Snellen chart (Snellen, 1862), the Mars Letter Contrast Sensitivity test (Arditi, 2005), and a version of the Stilling, Hertel, and Velhagen color panels test (Broschmann and Kuchenbecker, 2011), respectively. To select the hand with which participants would answer the tests, handedness was assessed using the Edinburgh Handedness Inventory (Oldfield, 1971). Additionally, subjects were screened for cognitive impairment and depression using the Mini-Mental State Examination (MMSE) (Folstein et al., 1975) and the Beck Depression Inventory II (BDI-II) (Beck et al., 1996). A score of ≤ 24 points in the MMSE and/or a score of ≥9 points in the BDI-II were considered exclusion criteria.

The subjects participated in a modified version of the image recognition task described in Miloserdov et al. (2020). Subjects were shown images from three different categories: cars, faces and scrambled images. The images were shown on two different contrast levels, high (100% contrast) and low (10% contrast), giving six different conditions in total. The subjects were asked to categorize each image they were shown. Each condition was repeated a total of 80 times, resulting in a total of 480 trials.

### 2.2. Measurement and Preprocessing of EEG Data

The EEG data was recorded at a sampling rate of 1,000 Hz using a 64-channel Brain Products system elastic cap. The cap includes a reference electrode located at FCz. The FieldTrip toolbox for Matlab (Oostenveld et al., 2010) was used for data preprocessing. Continuous EEG data was segmented into 1,500 ms long epochs (either 1,500 ms prestimulus or 1,500 ms poststimulus). What we will refer to as “raw” data was analyzed without going through any additional preprocessing steps.

What we will further refer to as the “preprocessed” data went through the following additional steps. An offline 0.1 Hz–220 Hz band-pass filter (butterworth, hamming window) and a 50 Hz notch filter were applied. Jumps and clips were automatically detected using a amplitude *z*-value cutoff of 20 in the case of jumps and a time threshold of 0.02 s for clips. The data points identified as clips or jumps were then linearly interpolated. Muscle artifacts were detected automatically by first applying a band-pass filter of 120 Hz–140 Hz and selecting an amplitude *z*-value threshold of 5. The trials marked as having muscle artifacts were afterwards visually inspected and rejected. Blink artifacts were corrected for using Independent Component Analysis (ICA). Data was re-referenced to the common average, i.e., the average across all EEG channels is subtracted from the EEG signal of each individual channel.

## 3. Methods

The aim of this study was to compare the results from classical frequency-based neuroscientific features as coherence and power spectra to features extracted on the basis of symbolic dynamics and information theoretical measures. From each domain, we took one single-channel measure and one functional connectivity measure that takes into account the relationships between different areas in the brain.

### 3.1. Functional Connectivity in EEG Recordings

Functional connectivity (FC) quantifies the temporal statistical dependence between signals in different brain regions (Sakkalis, 2011) and there exists an abundance of different measures that are used in the neuroscientific community. Generally, FC can be measured in time domain or frequency domain. In both domains, linear and non-linear measures exist. In this study, the non-linear time domain based measure mutual information (MI, Cover and Thomas, 1991) is compared to one of the most popular measures in EEG analysis, the frequency domain-based linear coherence (Bastos and Schoffelen, 2016). These two measures will be introduced in the following.

#### 3.1.1. Ordinal Pattern Statistics and Mutual Information

Ordinal patterns (OPs) are a symbolic approach to time series analysis that was originally introduced by Bandt and Pompe (2002). Since then, OP based methods have successfully been used in the analyses of biomedical data (Keller et al., 2007b; Amigó et al., 2010, 2015; Parlitz et al., 2012; Graff et al., 2013; Kulp et al., 2016; McCullough et al., 2017) and specifically EEG recordings (Keller et al., 2007a, 2014; Ouyang et al., 2010; Schinkel et al., 2012, 2017; O'Hora et al., 2013; Rummel et al., 2013; Shalbaf et al., 2015; Unakafov, 2015; Cui et al., 2016; Quintero-Quiroz et al., 2018). Statistics based on ordinal pattern have been shown to be robust to noise (Parlitz et al., 2012; Quintero-Quiroz et al., 2015) and can be used to define advanced concepts for quantifying information flow (Staniek and Lehnertz, 2008; Amigó et al., 2016) or to derive transition networks in state space from observed time series (McCullough et al., 2015; Zhang et al., 2017).

**Figure 1:**
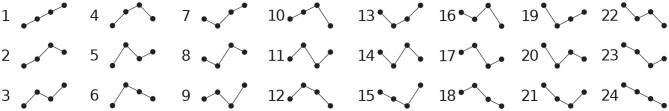
All 24 ordinal patters of length *w* = 4.

In ordinal pattern statistics, the order relations between values of a time series are considered rather than the values themselves. An ordinal pattern for a given length *w* and lag *l* describes the order relations between *w* points of a time series, each separated by *l* − 1 points. For a length *w*, there are *w*! possible different patterns, that can each be assigned a unique permutation index as illustrated for *w* = 4 in Figure 1. The permutation index characterizes the permutation π that is needed to get from a sample *x*_*t*_, *x*_*t*+*l*_, …, *x*_(*w*−1)__(*t*+*l*)_ of the time series to a sample *x*_π(*t*)_, *x*_π(*t*+*l*)_, …, *x*_π((*w*−1)__(*t*+*l*))_ that is ordered ascendingly according to the amplitude of the sample in the time series.

**Figure 2:**
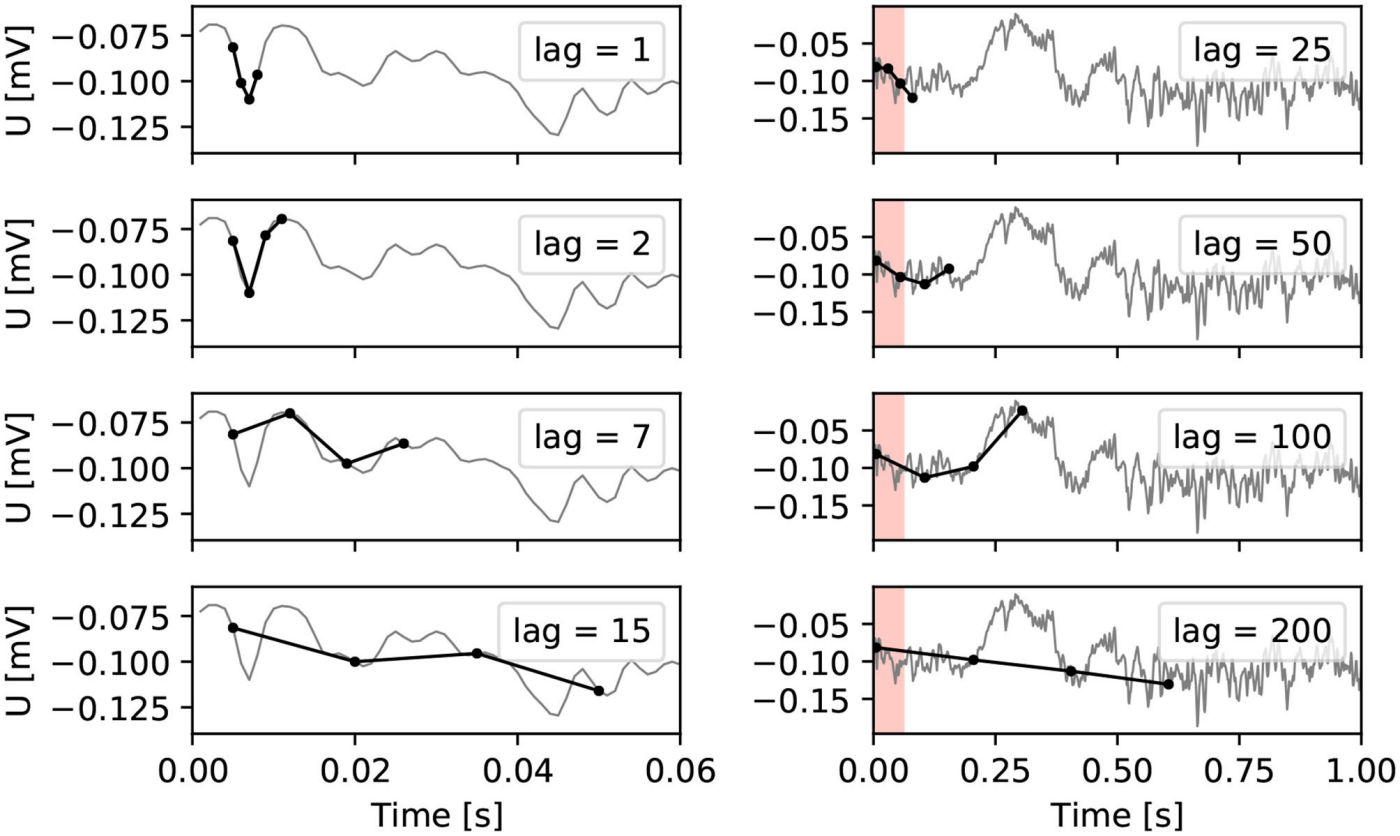
Illustration of ordinal patterns on different time scales in raw EEG data with sampling rate 1,000 Hz. The colored interval in the right column covers the same time span (0.06 s) as the entire window in the left column.

An important parameter is the lag *l* which can be used to address different time scales as illustrated in Figure 2.

Ordinal patterns are easy and inexpensive to compute and have been shown to be robust to noise (Bandt and Pompe, 2002; Parlitz et al., 2012). From a sequence of ordinal patterns, the probabilities of occurrence of specific patterns, given a lag *l* and length *w*, can be used to characterize the underlying time series. Commonly, complexity measures as permutation entropy (Bandt and Pompe, 2002; Parlitz et al., 2012) or conditional entropy (Unakafov, 2015) are applied to the resulting pattern distributions.

Here, the question asked is not about the complexity of a univariate time series, but about the similarity of channels in one multivariate EEG recording. The similarity measure that is used here is the mutual information (MI) (Shannon, 1948; Cover and Thomas, 1991). Mutual information can be expressed by the Kullback-Leibler divergence (Kullback and Leibler, 1951) between the joint probability distribution *p*_*X, Y*_ of two jointly varying random variables *X* and *Y* and the product of their marginal distributions:

(1) $$\begin{array}{l} I\left(X;Y\right)=\text{KL}\left(p_{X,Y}||p_{X}\cdot p_{Y}\right). \end{array}$$

For independent variables, the joint distribution is equal to the product of the marginal ones, resulting in a mutual information of *I*(*X*; *Y*) = 0. Accordingly, mutual information can be interpreted as a quantity that measures to what degree two random variables are not independent.

#### 3.1.2. Coherence

In contrast to OP statistics and MI, coherency (Bastos and Schoffelen, 2016) measures functional connectivity in the frequency domain. The *coherency* of two time series *y*_*i*_ and *y*_*j*_, for example two EEG channels *i* and *j*, is defined as the normed expectation value of the cross-spectrum

(2) $$\begin{array}{l} coh_{ij}\left(f\right)=\frac{\langle {ŷ}_{i}\left(f\right){ŷ}_{j}^{*}\left(f\right)\rangle }{\sqrt{\langle {ŷ}_{i}\left(f\right){ŷ}_{i}^{*}\left(f\right)\rangle \langle {ŷ}_{j}\left(f\right){ŷ}_{j}^{*}\left(f\right)\rangle }}, \end{array}$$

where ŷ_*i*_(*f*) is the Fourier transform of the signal *y*_*i*_(*t*) and ^*^ denotes the complex conjugate.

The expectation value 〈·〉 is usually approximated by taking the average over multiple trials from an EEG sample. In this study, we will consider the absolute value of Equation (2), and call it *coherence*.

### 3.2. Single-Channel Features in EEG Recordings

We compare the introduced FC measures to measures only taking into account single channels, but no relations between them. For this, we consider the PSDs as done by Suetani and Kitajo (2020), as well as OP distributions for each channel. In both cases, (not necessarily normalized) distributions per channel are considered. As a metric to compare them, we use the generalized KL-divergence. A vectorized, symmetric version of this was introduced by Suetani and Kitajo (2020) as a metric, which is given by

(3) $$\begin{array}{l} d_{nm}=\frac{1}{N_{\text{ch}}}\overset{N_{\text{ch}}}{\underset{l=1}{\sum }}\frac{1}{2}\left[D_{B}\left(S_{n}^{\left(l\right)}||S_{m}^{\left(l\right)}\right)+D_{B}\left(S_{m}^{\left(l\right)}||S_{n}^{\left(l\right)}\right)\right], \end{array}$$

where the generalized KL-divergence *D*_*B*_(*P*||*Q*) between two not necessarily normalized densities *P* and *Q* is given according to

(4) $$\begin{array}{l} D_{B}\left(P||Q\right)=\int \left(p\left(x\right)\log\frac{p\left(x\right)}{q\left(x\right)}-p\left(x\right)+q\left(x\right)\right)\text{d}x. \end{array}$$

*d*_*nm*_ gives the distance between two vectors of dimension *N*_ch_, where each dimension contains a distribution $S_{n}^{\left(l\right)}$, which will either be a PSD or a OP distribution. It is a special case of the beta-divergence (Basu et al., 1998; Mihoko and Eguchi, 2002) used in Suetani and Kitajo (2020) with β = 1.

### 3.3. Dimensionality Reduction

Dimensionality reduction aims to visualize such high-dimensional data in a low-dimensional space, preferably by extracting the most important features and representing each data point only by those. While the idea of dimensionality reduction dates back more than 100 years (Pearson, 1901), recently, more and more techniques have surfaced (van der Maaten et al., 2007).

In this study, the non-linear algorithm t-distributed stochastic neighbor embedding (t-SNE, van der Maaten and Hinton, 2008) is used to project features extracted from EEG time series. These features are adjacency matrices in case of functional connectivity and vectors of distributions in case of single-channel measures.

#### 3.3.1. Nonlinear Dimension Reduction (t-SNE)

T-distributed stochastic neighbor embedding was first introduced by van der Maaten and Hinton (2008) as an extension of stochastic neighbor embedding (SNE) (Hinton and Roweis, 2003) to avoid the crowding problem and simplify optimization.

The algorithm projects a set of *L* samples x_1_, x_2_, …, x_*L*_ from a high-dimensional into a low-dimensional space so that ${\mathbb{R}}^{N}∋{\text{x}}_{n}\mapsto {\text{y}}_{n}\in {\mathbb{R}}^{M}$, considering the so-called neighbor probabilities

(5) $$\begin{array}{l} p_{m|n}=\frac{\exp\left(-||{\text{x}}_{n}-{\text{x}}_{m}|{|}^{2}/\left(2\sigma_{n}^{2}\right)\right)}{\sum_{r\neq n}\exp\left(-||{\text{x}}_{r}-{\text{x}}_{n}|{|}^{2}/\left(2\sigma_{n}^{2}\right)\right)}. \end{array}$$

for high-dimensional data points **x**_*n*_ and **x**_*m*_. Here, ||·|| is typically the Euclidean norm. For projections of single-channel features, where one high-dimensional data point consists of *N*_ch_ distributions, we use ||·|| = *d*_*nm*_ in Equation (3). In case of connectivity matrices, we flatten^1^ the upper triangular part of the matrix and use the Euclidean norm as a measure of distance. *N* is an arbitrary integer value and *M* ∈ {2, 3} in general, we use *M* = 2 in this study. The probability *p*_*m*|*n*_ describes the probability that **x**_*m*_ is a neighbor of **x**_*n*_ and is proportional to a Gaussian centered at **x**_*n*_. The standard deviation σ_*n*_ of the high-dimensional probability distributions is calculated so that it satisfies a given value of the perplexity *k*. More specifically, the entropy of the conditional probability *p*_*m*|*n*_ as a function of *m* must be approximately equal to log_2_(*k*), or

(6) $$\begin{array}{l} k\left(p_{m|n}\right)=2^{H\left(p_{m|n}\right)}, \end{array}$$

where *H* is the Shannon entropy (Shannon, 1948). The goal of t-SNE is now to minimize the sum of the Kullback-Leibler divergences between the symmetric probabilities *p*_*nm*_ = (*p*_*m*|*n*_+*p*_*m*|*n*_)/2 in the high-dimensional space and the neighbor probabilities *q*_*nm*_ of the projections into low-dimensional space,

(7) $$\begin{array}{l} q_{nm}=\frac{\exp{\left(1+||{\text{y}}_{n}-{\text{y}}_{m}|{|}^{2}\right)}^{-1}}{\sum_{r\neq n}\exp{\left(1+||{\text{y}}_{r}-{\text{y}}_{n}|{|}^{2}\right)}^{-1}}, \end{array}$$

where *q*_*nm*_ is proportional to a Student-t-distribution (Student, 1908) with mean **y**_*n*_. The cost-function then becomes

(8) $$\begin{array}{l} C=\underset{n}{\sum }KL\left(P_{n}||Q_{n}\right)=\underset{n}{\sum }\underset{m\neq n}{\sum }p_{nm}\log\left(\frac{p_{nm}}{q_{nm}}\right). \end{array}$$

This cost-function is minimized using the gradient descent method, thus aligning the high- and low-dimensional neighbor probabilities. As a consequence, data points that are close in high-dimensional space will be projected closely together.

Because t-SNE is a stochastic method that starts out the projection with randomly assigning low-dimensional coordinates to data points and then minimizing the cost-function given in Equation (8), the resulting projection depends on the initial conditions of the projection. If one only considers a single projection, it is possible that this projection is not necessarily representative for all projections. Thus, if one wants to quantify effects in the t-SNE projections, one must average over many projections with different initial conditions.

### 3.4. Analysis Scheme

The EEG recordings were cut into trials. These trials were 1.5 s long, either directly before or directly after the subjects were shown a picture. These steps were done both for the raw and preprocessed versions of the data set.

OP sequences with patterns of length *w* = 4 were calculated for each trial. From all trials belonging to the same condition, histograms of the probability of occurrence were extracted, giving one histogram per EEG-channel, per condition and per subject. On the basis of the OP sequences, one single-channel feature and one functional connectivity feature were extracted:

- The marginal ordinal pattern distributions were used as single-channel features for dimensionality reduction with t-SNE. Here, we calculated the distance between two samples, each characterized by *N*_ch_ OP distributions, with Equation (3).
- We also used (joint) OP distributions to calculate MI-values between each pair of EEG channels, resulting in an *N*_ch_ × *N*_ch_ symmetric adjacency matrix per stimulus type per subject, containing the MI before or after each stimulus.

Additional to the time-domain measures, features were extracted from the frequency domain. For each trial, power spectra were calculated by performing a fast Fourier transform (FFT). Based on this, we again extracted one single-channel feature and one functional connectivity feature:

- The power spectral densities of each electrode were calculated as the squared absolute value of the Fourier transform of the signal and averaged over all trials. Again, we calculated the distance between two samples, each characterized by *N*_ch_ PSDs (either full PSD or only the bins of specific frequency bands), with Equation (2) where only the frequency bins of specific frequency bands were contained (alpha: 8 Hz–12 Hz, beta: 15 Hz–30 Hz, theta: 3 Hz–7 Hz, gamma: 30 Hz–50 Hz). We consider different frequency bands for comparability with findings in literature.
- Adjacency matrices based on the coherence between EEG channels were composed by calculating the average coherence over all trials per frequency-band.

The above described dimensionality reduction algorithms were applied to each feature set. In case of the adjacency matrices, the flattened upper triangular part of the matrices was used as input for the algorithms with the Euclidean distance as a metric. We also projected the vectors containing PSDs and OP distributions with the vectorized KL-divergence as distance measure as introduced in Equation (3).

It is important to mention that the different feature sets (FC and SC) involved in the comparison have different numbers of features which itself might influence dimensionality-reduction methods.

To quantify the effect of subject separation in the 2d-projections the ratio ρ between the average distances within a subject-cluster to the average distances to its three next neighbors was calculated. This ratio gives insight on how much closer data points of the same subject are projected together than data points of different individuals.

As a quantification of the separation of age groups we used a kernel density estimation (KDE; Silverman, 1986) with a Gaussian kernel and a bandwidth selection according to Scott (2015) for the distributions of the two age groups in the projections. An illustration of such estimated densities is given in **Figure 4**.

We then calculated the Jensen-Shannon divergence (Lin, 1991) between the two distributions. The Jensen-Shannon divergence quantifies differences between probability distributions. It is bound by unity in case of a base-2 logarithm and can be derived from the KL divergence via

(9) $$\begin{array}{lll} \text{JSD}\left(P||Q\right) & = & \frac{1}{2}\text{KL}\left(P||M\right)+\frac{1}{2}\text{KL}\left(Q||M\right),\;\text{where}\\ M & = & \frac{1}{2}\left(P+Q\right). \end{array}$$

The square root of the Jensen-Shannon divergence is a metric that is often called the Jensen-Shannon distance (JSD, Endres and Schindelin, 2003). This was done, in all cases, for 100 t-SNE projections of the ensemble with different random seeds.

## 4. Results

**Figure 3:**
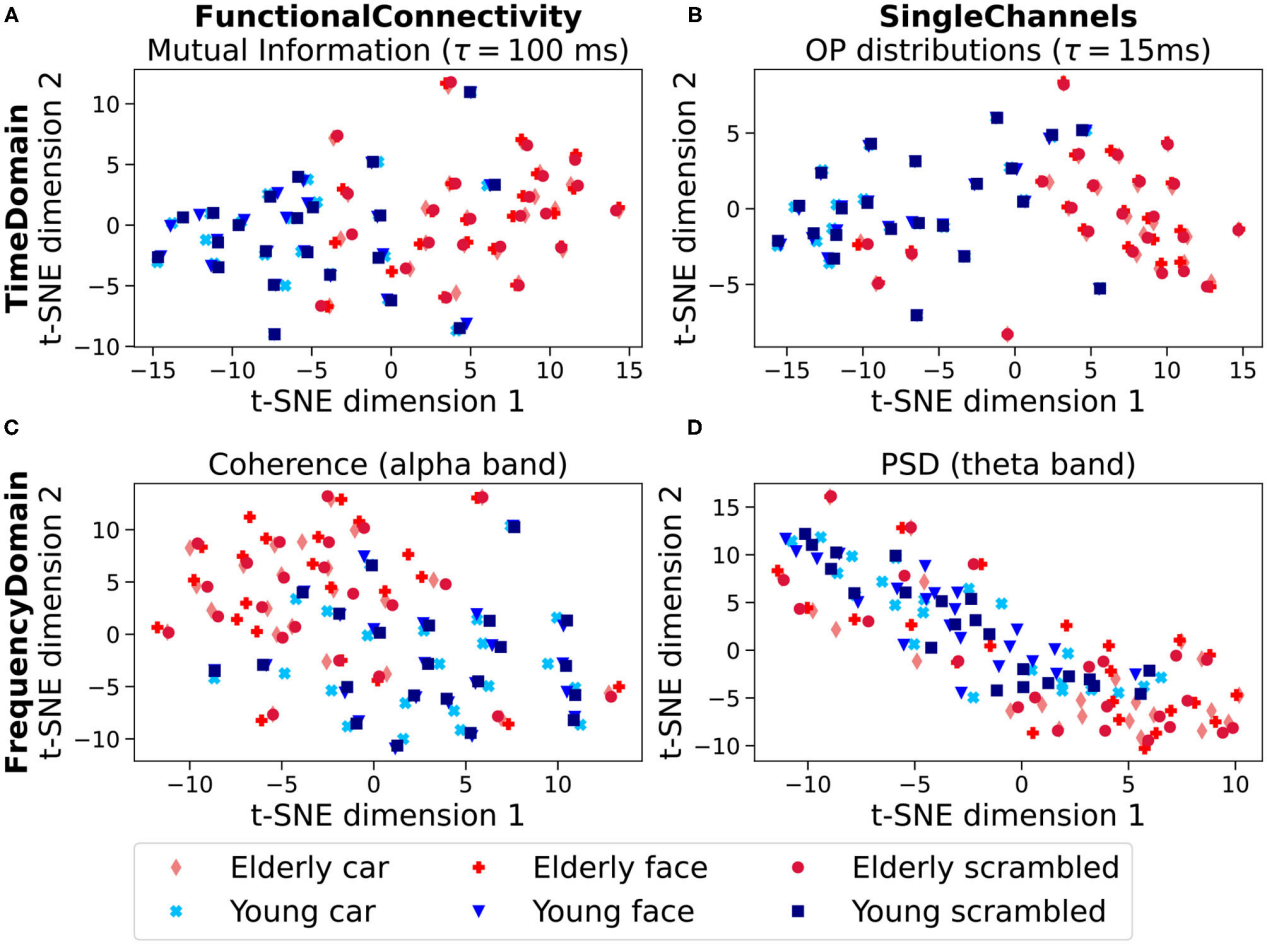
t-SNE projections of features obtained from the EEG time series of the Image Recognition data set (*k* = 30). We compare time-domain measures **(A,B)** to frequency domain measures **(C,D)**. The time-domain measures are extracted from raw data by calculating OP sequences, the frequency-domain measures are taken from frequency bands of the preprocessed data. In each case, a projection for the parameter (lag/frequency band) yielding the best group separation for the method is shown. **(A)** Projection of functional connectivity vectors obtained from OP statistics (τ = 100 ms), **(B)** Projection of OP distribution-vectors (τ = 15 ms) with the generalized KL-divergence as a metric, **(C)** Projection of functional connectivity vectors obtained from average coherence (alpha-band), **(D)** projection of power spectral density-vectors (theta-band) with the generalized KL-divergence as a metric.

**Figure A1:**
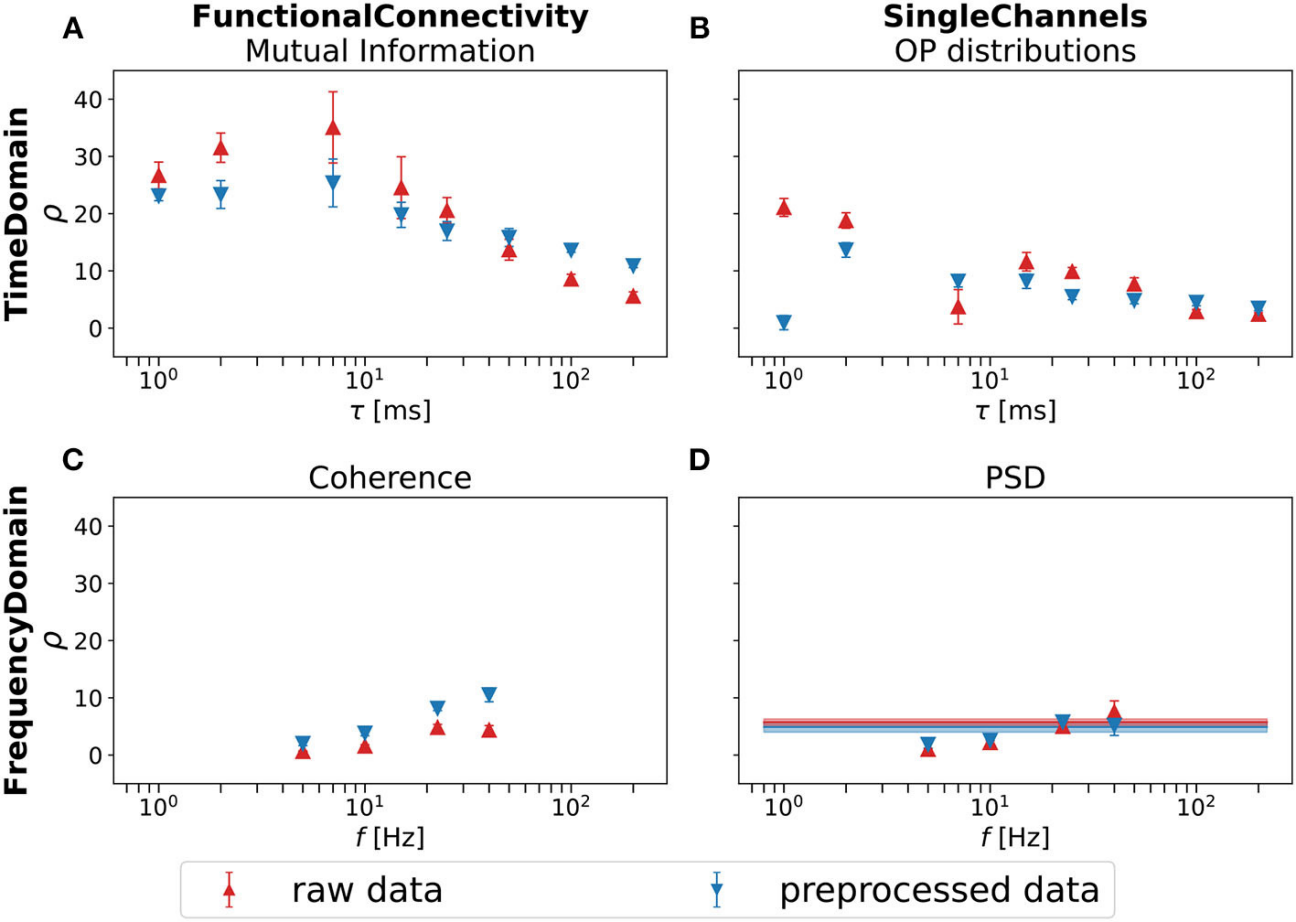
Quantification of separation of subjects in the t-SNE projections (perplexity *k* = 30) data set for raw and pre-processed EEG data. Both FC measures **(A,C)** and single-channel measures **(B,D)** were projected. For each projection, the ratio between the mean intra- and inter-cluster-distances was calculated. We call the Euclidean distances between points belonging to the same subject intra-cluster-distances, and the distances between the center of one cluster and its three next neighbors inter-cluster-distances. The error bars display the standard deviation over 100 t-SNE projections with different random seeds.

T-SNE projections of similar experimental conditions from the Image Recognition data set, i.e., samples after a high-contrast stimulus, are displayed in Figure 3. Four different feature types were projected. The results for the two FC features MI and Coherence (alpha band) are displayed in the left column, and the projections of the single-channel features, OP distributions and filtered PSDs (theta band), are depicted in the right column. In all cases, separations of age groups and individuals can be observed visually. We observe that the three conditions (face, car, scrambled image) are projected together closely for each individual. This effect is similar to the one observed in Suetani and Kitajo (2020) and is further quantified in **Figure A1** in the Appendix. The two age groups (Elderly in red and Young in blue) appear to be loosely separated.

As will be detailed in the following section we find that the separation of the two age groups, based on the JSD, are comparable for all feature sets, with a tendency toward higher separations for the OP based methods. The separation of individuals is clearly more distinct for OP based measures than for frequency based measures, both for FC and single channels.

### 4.1. Age Group Separation

**Figure 4:**
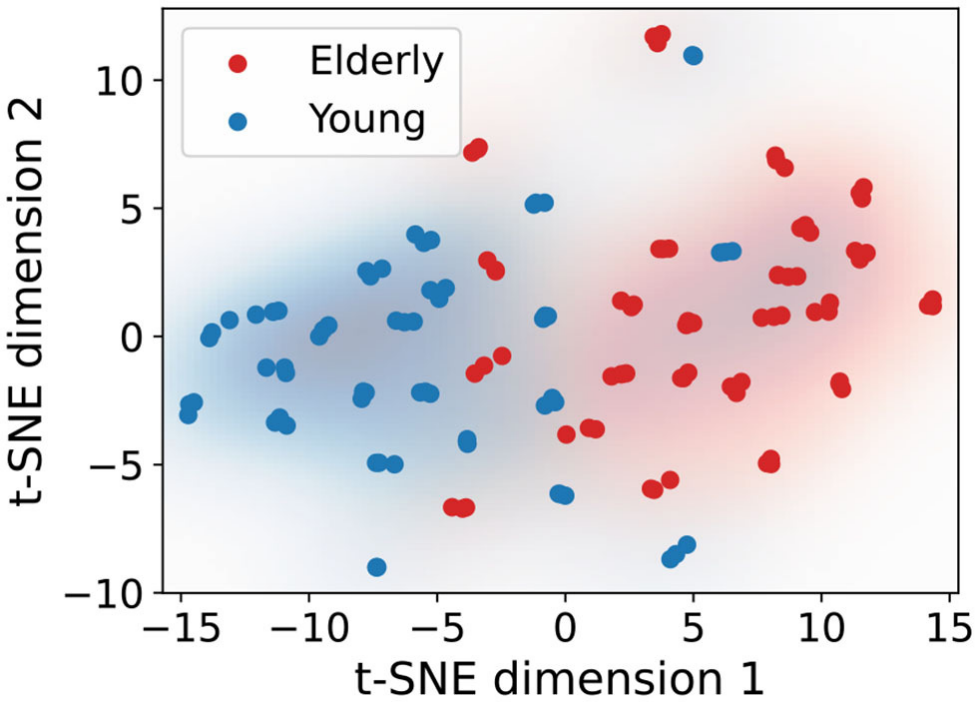
Exemplary plot of a the estimated kernel densities for the same parameters as in Figure 3A; a distinction is made only between age groups, not conditions.

In Figure 3, a separation of the two age groups in the t-SNE projections can be observed visually. As an example, in Figure 4, the estimated densities of the age groups are plotted for the same parameters as in Figure 3A. One can observe that the densities barely overlap.

**Figure 5:**
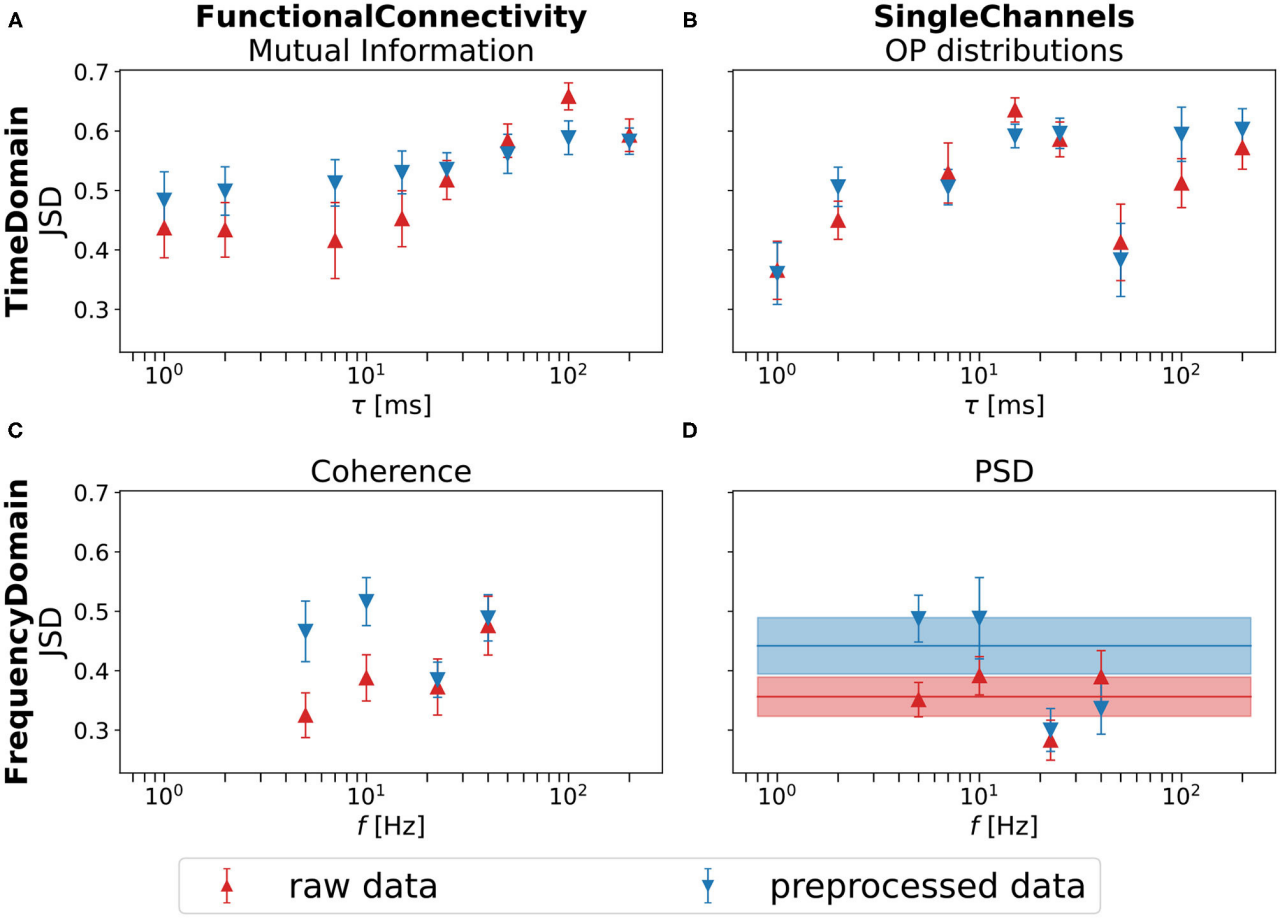
Quantification of the separation of Elderly and Young in 2d t-SNE projections (*k* = 30) for both raw (red) and preprocessed (blue) EEG data. We consider group separation based on **(A,C)** functional connectivity and **(B,D)** single channels. Results from OP based measures are displayed in the upper row, and from frequency-based measures in the lower row. The solid lines describe the separation when using the full PSD, otherwise only the frequency bins of one specific band are considered. The error bars display the standard deviation over 100 t-SNE projections with different random seeds.

The Jensen-Shannon distances between the two estimated distributions, depending on time or frequency scales, are displayed in Figure 5 for both raw and preprocessed data. For all feature sets, an average JSD larger than zero can be observed, with the highest values being achieved by the OP based measures.

While for the OP based measures, no increase of the best performance across time scales through artifact removal can be observed, artifact correction clearly leads to an overall increase of performance for frequency based measures. This is yet another illustration of the robustness of OP statistics.

We found no significant dependency of the separation of age groups on the perplexity *k* for the t-SNE projections. In case of individuals, we found the same dependency as Suetani and Kitajo (2020), where for larger values of *k* (up to *k* = 100), the separation is less distinct than for smaller values, but still observable.

## 5. Discussion

In this study, we obtained differences between age groups (Elderly, Young) and individuals subjected to similar experimental conditions based on both functional connectivity and single-channel measures obtained from multichannel EEG time series. We found that t-SNE as a method for dimensionality reduction and feature extraction does not only reflect individuality but also appears to represent inter-individual relationships, given by age groups in this case.

It should be emphasized that the separation of individuals is restricted to the separation of recordings from the same individual under similar conditions (post high contrast stimulus). Separate checks using pre-stimulus recordings and resting state recordings from the same session, revealed that the data points of the same individual are not necessarily projected closely together.

Regarding the separation between age groups, one could consider that a difference between brain age, which is a descriptor of the physiological condition of the brain, and the chronological age of the subject has been hypothesized (Irimia et al., 2015; Steffener et al., 2016). If the chronological age and the brain age of some individuals in the study differ, this could explain apparent outliers in the projections.

Since the aim of t-SNE is to find a low-dimensional projection of a high-dimensional data set that represents the distances between high-dimensional points, it can be assumed that the projected ensemble does not only represent the two features with highest variance and omits the others, but is rather a representation of the whole feature set.

We showed that OP analysis can obtain results comparable to classical EEG feature sets, and even outperform them if both methods are applied to raw data. For the OP based measures, the applied preprocessing pipeline partially even leads to a decrease in performance regarding the separation of age groups, while for the frequency-based measures, there is always an increase observable. This supports previous observations that OP based methods yield promising results if applied to raw data sets. Preprocessing could thus be reduced to avoid potential differences of analyses in different labs.

The question answered here is “Is the average or best performance of one feature set comparable to the average or best performance of another feature set?” This appears to be the case for the age group separation, and also for the separation of subjects.

Given the observation that there appears to be no significant difference between FC and SC measures, the question arises whether the obtained functional networks actually contain the information that we assume they do. In light of these findings, the interpretation of other studies that obtained age group differences based on functional connectivity (Wada et al., 1998; McIntosh et al., 2013; Al Zoubi et al., 2018, amongst others) could be reconsidered.

If the age differences are also contained in single-channel measures, and the separation power of functional connectivity does not outperform the single channel measures, it must be thoroughly investigated how these group differences are related to one another.

A possible explanation for the lack of differences between functional connectivity and single-channel measures would be that the main information that is contained in the functional connectivity measures that are considered here is due to shared sources between the different EEG channels. This information would also be contained in single-channel features. To verify this, further tests must be done.

Furthermore, future studies should include a comparison with other measures of network physiology like time delay stability (Bartsch et al., 2015; Liu et al., 2015; Lin et al., 2016) and transfer entropy (Schreiber, 2000; Staniek and Lehnertz, 2008; Vicente et al., 2011; Wibral et al., 2013) measures.

## Data Availability Statement

The datasets presented in this article are not readily available due to data protection rules. Requests to access the datasets should be directed to Melanie Wilke (melanie.wilke@med.uni-goettingen.de).

## Ethics Statement

The studies involving human participants were reviewed and approved by medical ethics committees of the University Medical Center Göttingen, Germany. The patients/participants provided their written informed consent to participate in this study.

## Author Contributions

IK, AS, and UP prepared the design of the manuscript. MW and DT-T designed the behavioral tasks. DT-T performed the experiments and was responsible for data acquisition. DT-T and IS performed data preprocessing. AS, IK, SB, UP, and IS developed the conceptional design of the data analysis. IK and SB implemented the analysis algorithms and performed the data analysis. MB, SL, and MW were in charge of project administration and funding acquisition. All authors edited and reviewed the article and approved the manuscript.

## Conflict of Interest

The authors declare that the research was conducted in the absence of any commercial or financial relationships that could be construed as a potential conflict of interest.

## Footnotes

^1^We “flatten” a matrix by concatenating all column vectors into one long vector.

## Appendix

To quantify the effect of subject separation and its dependency on considered time scales, the ratio ρ of intra- and inter-cluster-distances was calculated on different time scales. For each projection, the ratio between the mean intra- and inter-cluster-distances was calculated. The average ratio ρ and the standard deviation, depending on the lag for the OPs or the frequency band, is displayed in Figure A1.
